# Function of Protein S-Palmitoylation in Immunity and Immune-Related Diseases

**DOI:** 10.3389/fimmu.2021.661202

**Published:** 2021-09-07

**Authors:** Yuqi Zhang, Ziran Qin, Wenhuan Sun, Feng Chu, Fangfang Zhou

**Affiliations:** Zhejiang University City College, Hangzhou, China

**Keywords:** palmitoylation, acylation, immunity, immunologic diseases, palmitoyltransferases

## Abstract

Protein S-palmitoylation is a covalent and reversible lipid modification that specifically targets cysteine residues within many eukaryotic proteins. In mammalian cells, the ubiquitous palmitoyltransferases (PATs) and serine hydrolases, including acyl protein thioesterases (APTs), catalyze the addition and removal of palmitate, respectively. The attachment of palmitoyl groups alters the membrane affinity of the substrate protein changing its subcellular localization, stability, and protein-protein interactions. Forty years of research has led to the understanding of the role of protein palmitoylation in significantly regulating protein function in a variety of biological processes. Recent global profiling of immune cells has identified a large body of S-palmitoylated immunity-associated proteins. Localization of many immune molecules to the cellular membrane is required for the proper activation of innate and adaptive immune signaling. Emerging evidence has unveiled the crucial roles that palmitoylation plays to immune function, especially in partitioning immune signaling proteins to the membrane as well as to lipid rafts. More importantly, aberrant PAT activity and fluctuations in palmitoylation levels are strongly correlated with human immunologic diseases, such as sensory incompetence or over-response to pathogens. Therefore, targeting palmitoylation is a novel therapeutic approach for treating human immunologic diseases. In this review, we discuss the role that palmitoylation plays in both immunity and immunologic diseases as well as the significant potential of targeting palmitoylation in disease treatment.

## Introduction

Post-translational modifications (PTMs), such as acetylation, ubiquitination, methylation, and lipidation, can alter the structure, chemical properties, and function of proteins (1). These modifications are essential for information exchange and maintenance of homeostasis in cells and organisms. Protein lipidation is an important and diverse class of PTM. Lipidated proteins show high nonpolarity, which enhances the regulation of their localization and fluidity within the cell (2). Prenylation and fatty acylation are prevalent forms of lipid modifications (3). Prenylation is a post-translational attachment of a prenyl group, a 15-carbon farnesyl or a 20-carbon geranylgeranyl, to a conserved cysteine residue at or near the C-terminus CAAX motif (C: cysteine, A: aliphatic amino acids, X: any amino acid) (4). Fatty acylation includes two commonly recognized forms, N-myristoylation and palmitoylation. Protein N-myristoylation is a co-translational attachment of a 14-carbon myristic acid to the alpha amino group of an N-terminal glycine residue through a stable amide linkage. Conspicuously, the N-terminal of all myristoylated proteins contains the common sequence methionine-glycine-X-X-X-serine/threonine (M-G-X-X-X-S/T; X: any amino acid) and is amenable to N-myristoylation (5, 6). There are three main types of palmitoylation: S-, N- and O-palmitoylation according to the chemical linkage by which the fatty acid is attached to the protein. Among these, N-palmitoylation can occur at both the protein N-terminus and the epsilon amino group of specific lysine residues with the palmitate group connected to the protein *via* an amide bond. S-palmitoylation occurs on cysteine residues, involves a thioester linkage and is a reversible modification. O-palmitoylation involves the attachment of palmitate to serine and threonine residues *via* an ester bond (7–10).

Lipid modifications can promote membrane-association to otherwise soluble proteins (11). However, palmitoylation is more than just a simple membrane anchor. Palmitoylation significantly increases the hydrophobicity of proteins, leading to changes in their conformation, stability, intracellular transport, localization and binding affinity to cofactors (3, 12). Many proteins require palmitoylation for their correct folding and proper structure. Mutation of the palmitoylation site may lead to erroneous protein synthesis and ubiquitination-mediated protein degradation (13). Trafficking of membrane-associated proteins from the early secretory site to the appropriate cellular destination is, in many cases, dependent on palmitoylation (14–16). Palmitoylation also plays a critical role in regulating protein-protein interactions, which ensures proper signal transduction (17–20).

Most lipidation events, including N-myristoylation, N- and O-palmitoylation, are essentially irreversible. In contrast, protein S-palmitoylation is a reversible and dynamically regulated post-translational modification. The labile thioester bond allows proteins to cycle between palmitoylated and de-palmitoylated forms in a time frame of seconds to hours (21–24). The reversible nature of S-palmitoylation makes it a significant lipid modification in terms of controlling protein function. For other excellent recent reviews of S-palmitoylation in immune signaling, the reader is referred to refs. (25, 26). In this review, we describe the process of S-palmitoylation, introduce palmitoyl acyltransferases, acyl protein thioesterases and elaborate on the role of S-palmitoylation in immune regulation. Moreover, the therapeutic potential of targeting S-palmitoylation in immunologic diseases is discussed.

## Protein Palmitoylation and Depalmitoylation

S-palmitoylation refers to the addition of palmitate to cysteine residues. However, palmitoleate, stearate, and oleate, as well as long-chain polyunsaturated fatty acids such as arachidonate and eicosapentaenoate can also be post-translationally linked to one or more cysteine residues *via* thioester (S-acyl) bonds. Therefore, the more general terms “thioacylation” or “S-acylation” are often used instead of palmitoylation. In this review, palmitoylation refers to the attachment of palmitate and other long-chain fatty acids to cysteine residues *via* thioester. It is estimated that more than 9000 proteins of commonly studied organisms such as human and mice can undergo palmitoylation, and more than 1000 palmitoylation has been discovered (27). Dynamic palmitoylation is under the tight control of two opposing types of enzymes. Palmitoyl acyltransferases (PATs) catalyze the attachment of palmitoyl groups, while the removal of thioester-linked long-chain fatty acids from cysteine residues is mediated by acyl protein thioesterases (APTs) (28, 29).

### Protein Palmitoylation and PATs

**Figure 1:**
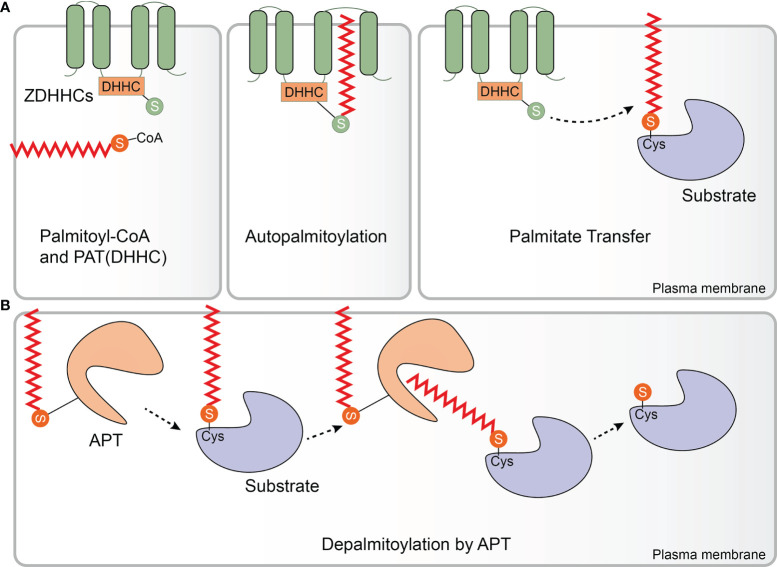
Protein palmitoylation and depalmitoylation. **(A)** Palmitate (from palmitoyl-coenzyme A (CoA)) can be attached to substrate proteins *via* a thioester linkage by Zinc finger aspartic acid-histidine-histidine-cysteine domain-containing protein (zDHHC)-palmitoyl acyltransferases (PATs). zDHHC-PATs are integral membrane proteins (green) that typically have 4 transmembrane domains. The enzyme’s catalytic aspartic acid-histidine-histidine-cysteine (DHHC) motif is located in the cytoplasm. zDHHC-PATs first undergo an autopalmitoylation event before the palmitate group is transferred from the cysteine residue of the DHHC motif to a specific cysteine on a substrate protein (purple). **(B)** Enzymatic removal of the fatty acid groups from palmitoylated proteins (purple) is catalyzed by acyl protein thioesterases (APTs), a family of serine hydrolases including the enzymes APT1/2 (orange). APT1/2 are themselves palmitoylated which anchors them to the membrane giving them ready access to the fatty acid which is to be removed from the substrate protein. APT1/2 both have a hydrophobic pocket in which the palmitate group binds prior to its cleavage. Following hydrolysis of the thioester bond, the fatty acid is released from the substrate and diffuses into the membrane. (Palmitate is the approximate height of one leaflet of the lipid membrane, here it is enlarged to highlight.).

PATs were first identified and isolated from *Saccharomyces cerevisiae*. Effector of Ras function 2 (Erf2) was the first enzyme identified to catalyze protein palmitoylation. *ERF2* encodes a 41-kDa integral membrane protein harboring a motif consisting of the amino acids aspartic acid-histidine-histidine-cysteine (DHHC) within a cysteine-rich domain (CRD). Erf2 is essential for the palmitoylation and proper subcellular localization of Ras proteins. Deletion of *ERF2* resulted in a decrease in Ras2 palmitoylation and plasma membrane association. In addition, the DHHC-CRD motif is required for Erf2 function (30). Six other DHHC-containing proteins, Akr1, Akr2, Swf1, Pfa3, Pfa4, and Pfa5, were then discovered by screening the yeast genome, and most of them catalyze S-palmitoylation (31–35) Although the enzymes that catalyze protein palmitoylation were discovered in 1999 (30), the molecular basis of their catalytic activity was not determined for another decade. In 2010, Mitchell and colleagues examined the molecular mechanism of palmitate transfer of the yeast Ras PAT by combining genetic and biochemical methods, and found that the palmitoylation reaction occurs in a two-step manner consisting of autopalmitoylation of the DHHC containing enzyme to create a palmitoyl-Erf2 intermediate, followed by transfer of the palmitoyl group to Ras2 (**Figure 1A**). Palmitoyl-coenzymeA (CoA) was found to be the palmitate donor. It is worth noting that the first step of the catalytic reaction takes place in a matter of seconds. Furthermore, mutational analysis indicated that the DHHC motif serves as the catalytic center of the PAT enzyme (36). This finding was subsequently confirmed in a series of experiments carried out by Jennings and co-workers who developed a high-performance liquid chromatography (HPLC) based method for studying the activity of mammalian DHHC2 and 3. In these experiments, it was shown that Zinc finger DHHC domain-containing protein (zDHHC)-PATs first acylate themselves in the presence of palmitoyl CoA before the fatty acid group is transferred to a substrate protein, which is consistent with a two-step ping-pong kinetic transfer mechanism (37). It was also shown that zDHHC2 and 3 had different preferences for acyl-CoAs of differing chain length which was the first time that zDHHC-PAT lipid co-factor specificity had been observed (37).

All zDHHC-PATs share a common DHHC motif to mammals, and is the catalytic core of the enzyme. zDHHC-PATs are integral membrane proteins and typically have four transmembrane domains (TMD) with both the N- and C-termini exposed to the cytoplasm (38). The DHHC motif is located with the 51 amino acid CRD which is located between the second and third TMDs (39, 40). The palmitoyl transfer reaction occurs on the cysteine residue of the DHHC motif (41, 42). This cysteine is also involved in coordinating two zinc atoms, which do not play a catalytic role but are responsible for proper folding and stability of zDHHC-PATs (43, 44). To date, 23 zDHHC-PATs have been identified in mammals, designated as zDHHC21 to zDHHC24 omitting zDHHC10 (28). zDHHC-PATs have different cellular locations with the majority of zDHHC-PATs resident in either the endoplasmic reticulum (ER) and/or Golgi, with zDHHC5, 20 and 21 positioned in the plasma membrane although it should be noted that some zDHHC-PATs may be located in different membrane compartments in various cell types (45–48). Recently, the first crystal structure of a mammalian zDHHC-PAT (human zDHHC20) was determined. The four TM helices of zDHHC20 adopt a tepee-like organization in the membrane, and the residues lining this cavity determine the acyl-CoA chain length selectivity of the enzyme. The TM helices are connected by short loops between TM1 and TM2 as well as between TM3 and TM4 whereas the extended DHHC-CRD region is located between TM2 and TM3 (40). In addition to having different preferences for acyl-CoA chain length, zDHHC-PATs display different preferences for different substrate proteins (49–51). However, as no consensus palmitoylation sequence motifs have yet been identified, the mechanism of zDHHC-PAT substrate specificity is only poorly understood at present (52). This said, the cytoplasmic facing ankyrin-repeat domains of zDHHC13 and 17 are involved in determining the substrate specificity of these enzymes (53, 54). Some zDHHC-PATs require accessory proteins for their activity, and are essential for protein stability, plasma membrane stabilization and substrate selectivity (55–58). For some substrate proteins, an earlier initial lipidation event is required before they can be palmitoylated e.g., Ras (59–61).

### Protein Depalmitoylation and APTs

Compared with the large number of zDHHC-PATs which are known, only several APTs have been identified so far, including palmitoyl‐proteinthioesterase‐1 (PPT1), PPT2, APT1, APT2, α/β hydrolase domain 17 (ABHD17A/B/C) and ABHD10 (62–69). Interestingly, the first APT was identified before zDHHC-PATs were discovered when in 1993 PPT1 was isolated from bovine brain cytosol (62). PPT1 is mainly localized to lysosomes and is critical for the depalmitoylation of proteins during the process of protein degradation (62). In conventional type 1 dendritic cells (cDC1s), PPT1 is highly expressed and acts as a protective molecular rheostat in anti-viral immunity. PPT1 protects steady state cDC1s from viruses by facilitating antigen degradation and endosomal acidification *via* V-ATPase recruitment. After cDC1s activation, PPT1 is immediately downregulated to promote efficient cross-presentation, costimulatory molecules and inflammatory cytokine production (70). PPT2 is another lysosomal thioesterase that shares a degree of amino acid sequence similarity with PPT1 (63). However, compared to PPT1, PPT2 has a smaller lipid-binding pocket, which contributes to differences in their substrate specificity. PPT1 preferentially catalyzes the removal of thioester-linked long-chain fatty acids from palmitoylated substrates whereas PPT2 prefers substrate proteins to which palmitoyl-CoA is attached (20). More recently, APT1 has been reported as having thioesterase activity (64). APT1 can self-depalmitoylate at Cys2, and contains a hydrophobic pocket within which it can accept the fatty acids that are to be cleaved off the acylated protein, potentially resulting in its own membrane-cytosol shuttling and steady-state regulatory (71) (**Figure 1B**). APT2 exhibits high amino acid sequence and structure similarity to APT1. Although APT2 can also catalyze self-depalmitoylation at Cys2, APT1 and 2 have distinct substrate specificity although why this should be is not yet understood (66, 71–73). Furthermore, APT1 is primarily localized to mitochondria while APT2 is mainly found in the cytoplasm (74). Although soluble APT2 is vulnerable to proteasomal degradation, membrane binding can protect APT2 from this as well as provide it with sufficient time to encounter its membrane-associated target substrates. Mechanistically, APT2 can associate with membranes in three different ways: through electrostatic attraction, insertion of a hydrophobic loop and through palmitoylation by either zDHHC3 or 7. Once bound to the membrane, APT2 is predicted to deform the lipid bilayer and trigger extraction of the acyl chain to capture it in its hydrophobic pocket (75). In addition, Kathayat et al. developed a fluorescent probe for S-depalmitoylation activity to monitor the endogenous activity levels of APTs and confirmed that APTs activity is dynamically regulated (76). Somewhat surprisingly, the proteomic profiling of dynamic protein palmitoylation revealed that the majority of proteins are not dynamically regulated by APTs at all. The enzymatically regulated dynamic palmitoylation events exhibit markedly different turnover rates and are targeted to specific proteins with annotated roles in particular in cell growth, migration, and cancer, indicating that serine hydrolase-mediated turnover of protein palmitoylation is not a general phenomenon (24). The ABHD17 family of enzymes have been shown to have potent thioesterase activity as well. ABHD17 enzymes are mainly found in the plasma membrane and contribute to N-Ras and synaptic protein depalmitoylation (67). It is thought that ABHD17 enzymes are palmitoylated at their N-terminal cysteine cluster, and that this is required for their association with the plasma membrane and interactions with substrates (77). Additionally, ABHD10, a mitochondrial resident protein, was recently shown to have thioesterase activity. ABHD10 has an extensive “cap” domain and possesses slower kinetic parameters than APT1 *in vitro.* Research into the functional properties and depalmitoylation mechanisms of ABHD10 is in its infancy (69). In short, protein depalmitoylation is carried out by an ever increasing diverse range of thioesterase enzymes that have different catalytic properties and substrate specificities. PATs and APTs interconnect through complex regulatory networks, having crucial implications for the regulation of substrates.

## Emerging Roles for Palmitoylation in Innate Immunity

As the first line of host defense against invading pathogens, innate immunity represents an evolutionary response that enables host survival. The strategy for pathogen recognition is based on host germline-encoded pattern recognition receptors (PRRs) identifying unique pathogen-associated molecular patterns (PAMPs). Endolysosomes and the cytosol are the two main sites for PAMP detection. Therefore, the innate immune response is initiated by distinct innate immune sensors: endosomally localized transmembrane Toll-like receptors (TLRs) and C-type lectin receptors (CLRs), and cytosolic nucleotide-binding oligomerization domain (NOD)-like receptors (NLRs) (DNA and RNA sensors). These sensors ensure a rapid immune response to low-level stimuli (78). In the last few years, many advances have been made in our knowledge about the role palmitoylation plays in innate immunity. Here, we provide an overview of recent research progress related to the function of palmitoylation in innate immunity.

### Palmitoylation and the TLR Signaling Pathway

**Figure 2:**
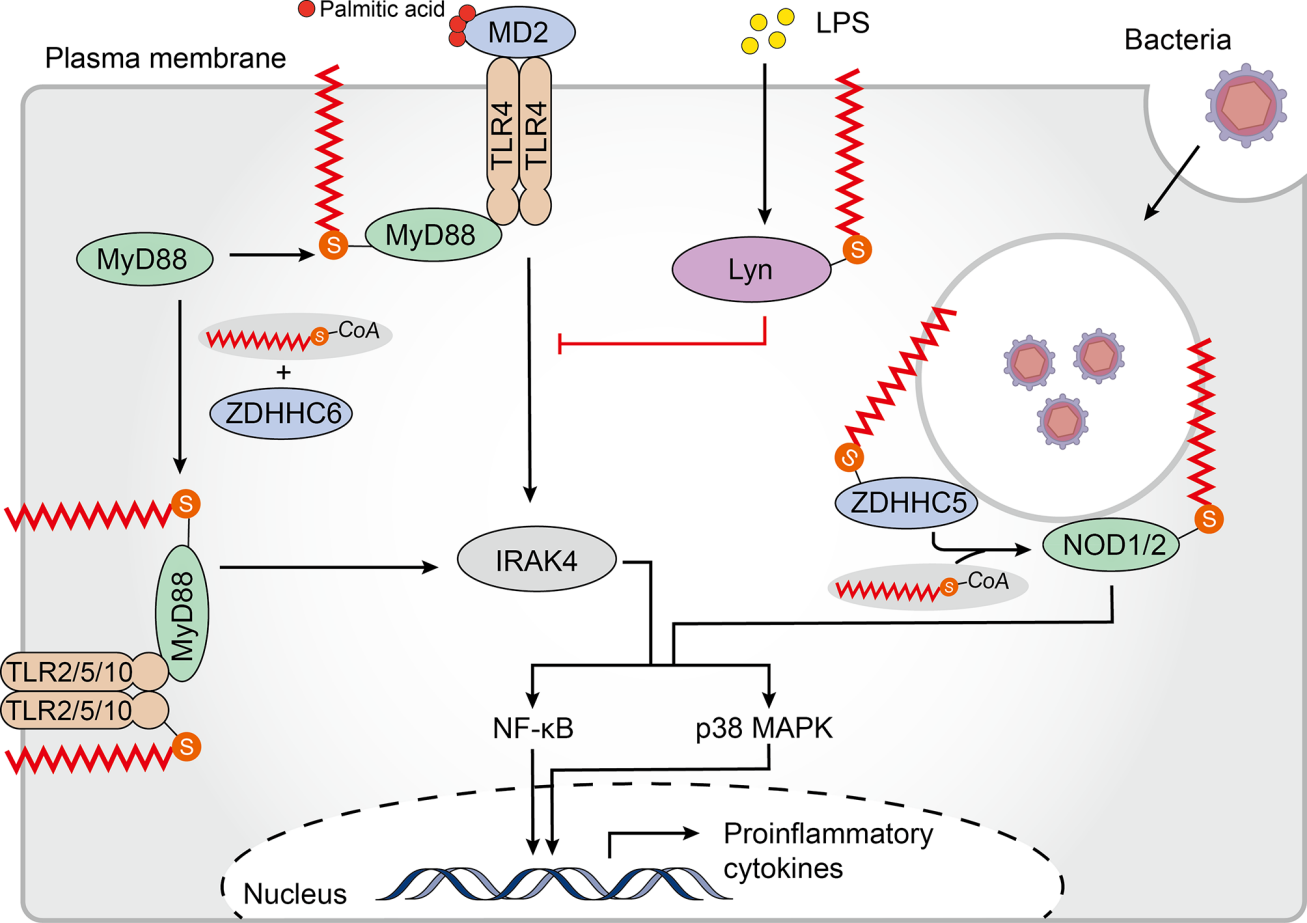
Palmitoylation in the TLR and NLR pathways. Membrane-bound Toll-like receptors (TLRs)-2/5/10 are all palmitoylated. Palmitoylation of TLR2 targets it to the plasma membrane where it interacts with myeloid differentiation primary response protein (MyD88), which regulates the nuclear factor kappa B (NF-κB) and p38-mitogen-activated protein kinase (MAPK) signaling pathways. Binding of MyD88 to interleukin-1 receptor-associated kinase 4 (IRAK4) and downstream signal activation requires palmitoylation of MyD88 by Zinc finger aspartic acid-histidine-histidine-cysteine domain-containing protein (zDHHC) 6. Although TLR4 is not palmitoylated, palmitic acid binds to its accessory protein myeloid differentiation protein 2 (MD2) to promote pro-inflammatory signaling. Furthermore, lipopolysaccharide (LPS) induces palmitoylation of Lyn kinase, which downregulates TLR4 pro-inflammatory signaling. Palmitoylation of cytosolic nucleotide oligomerization domain-like receptors 1 and 2 (NOD1/2) drives their translocation to bacteria-containing endosomes where they function in signal transduction. zDHHC5 is also recruited to the endosomes that contain bacteria where it palmitoylates both NOD 1 and 2.

TLRs, the first PRRs to be identified and the main integral membrane PRRs, play a central role in recognition of components derived from a wide range of pathogens and initiation of innate immune responses (79). To date, 10 human TLRs localized on the cell surface (TLR1/2/4/5/6/10) or within intracellular vesicles (TLR3/7/8/9) have been identified to recognize distinct sets of pathogen-associated molecules (80). The human TLR2 is palmitoylated primarily at Cys609 by zDHHC 2, 3, 6, 7, and 15. In primary cultured dendritic cells (DCs), palmitoylation-deficient TLR2 was less able than wild-type TLR2 to induce nuclear factor kappa B (NF-κB)-dependent gene expression. Additionally, treatment with the non-specific zDHHC-PAT covalent inhibitor 2-bromopalmitate (2-BP) significantly impaired proinflammatory cytokine production in response to TLR2 microbial ligands, indicating that palmitoylation of TLR2 was necessary for inducing TLR2-mediated pro-inflammatory signaling. Furthermore, palmitoylation promotes cell surface localization of TLR2, and likely affects interactions with its ligands. Besides TLR2, the flagellin receptors TLR5 and 10 have also been shown to be palmitoylated (81). Whether and how palmitoylation regulates TLR expression, localization and function remains to be addressed. Of note, although the bacterial lipopolysaccharide (LPS) receptor TLR4 is not palmitoylated, palmitic acid, which binds directly to the TLR4 accessory myeloid differentiation protein 2 (MD2) is required to trigger its pro-inflammatory signaling (82, 83). However, LPS induced accumulation of palmitoylated Lyn kinase in lipid rafts can downregulate TLR4 pro-inflammatory signaling (84) (**Figure 2**). Further studies are needed to reveal the mechanism and impact of palmitoylation of TLRs on recognizing and resisting pathogens.

The TLR signaling pathway originates from the intracytoplasmic toll-interleukin 1 receptor (TIR) domains, which are conserved in all TLRs. TIR domain-containing adapters, such as the myeloid differentiation primary response protein (MyD88), modulate TLR signaling pathways (85). MyD88 can be palmitoylated at Cys113 and Cys274 by zDHHC6 using endogenous fatty acids synthesized by a fatty acid synthase (FASN) or exogenous fatty acids supplied by CD36-mediated uptake. Notably, binding of MyD88 to interleukin-1 receptor-associated kinase 4 (IRAK4), and activation of p38 mitogen-activated protein kinase (MAPK) and NF-κB p65 were significantly impaired in MyD88 where Cys113 was replaced with alanine, indicating the significance of Cys113 palmitoylation in facilitating TLR signaling. Moreover, decreased inflammatory responses were observed after treatment with FASN inhibitors or zDHHC6 knockdown (86) (**Figure 2**). These findings suggest that palmitoylation of TLRs and their adapters is a potent factor regulating the TLR-mediated pro-inflammatory responses.

### Palmitoylation and the NLR Signaling Pathway

Pathogens that escape endosomal detection and invade the cytosol can be detected by cytosolic sensors. NLR proteins are an important class of cytoplasmic PRRs that play a key role in microbial sensing, thereby triggering antimicrobial immune responses (87). Although soluble in the cytosol, NOD‐like receptors 1 and 2 (NOD1/2) require recruitment to bacteria-containing endosomes to elicit an efficient signaling response (88–91). Biologists have been working to establish an accurate model to describe how NOD proteins are recruited to intracellular membranes and mediate ligand sensing. Recently, Lu and colleagues have shown that targeting of both NOD1 and NOD2 to bacteria-containing endosome membranes requires their palmitoylation by zDHHC5, where they recognize pathogenic bacterial-derived peptidoglycan components in the cytoplasm promoting intracellular NOD1/2 mediated immune responses including autophagy and release of inflammatory factors (92). However, the exact mechanism of zDHHC5 recruitment to the site of bacterial entry is not yet known. This paper elegantly demonstrates the relationship between dynamic palmitoylation and regulation of NLRs in antimicrobial responses.

### Function of Palmitoylation in Sensing DNA and RNA

**Figure 3:**
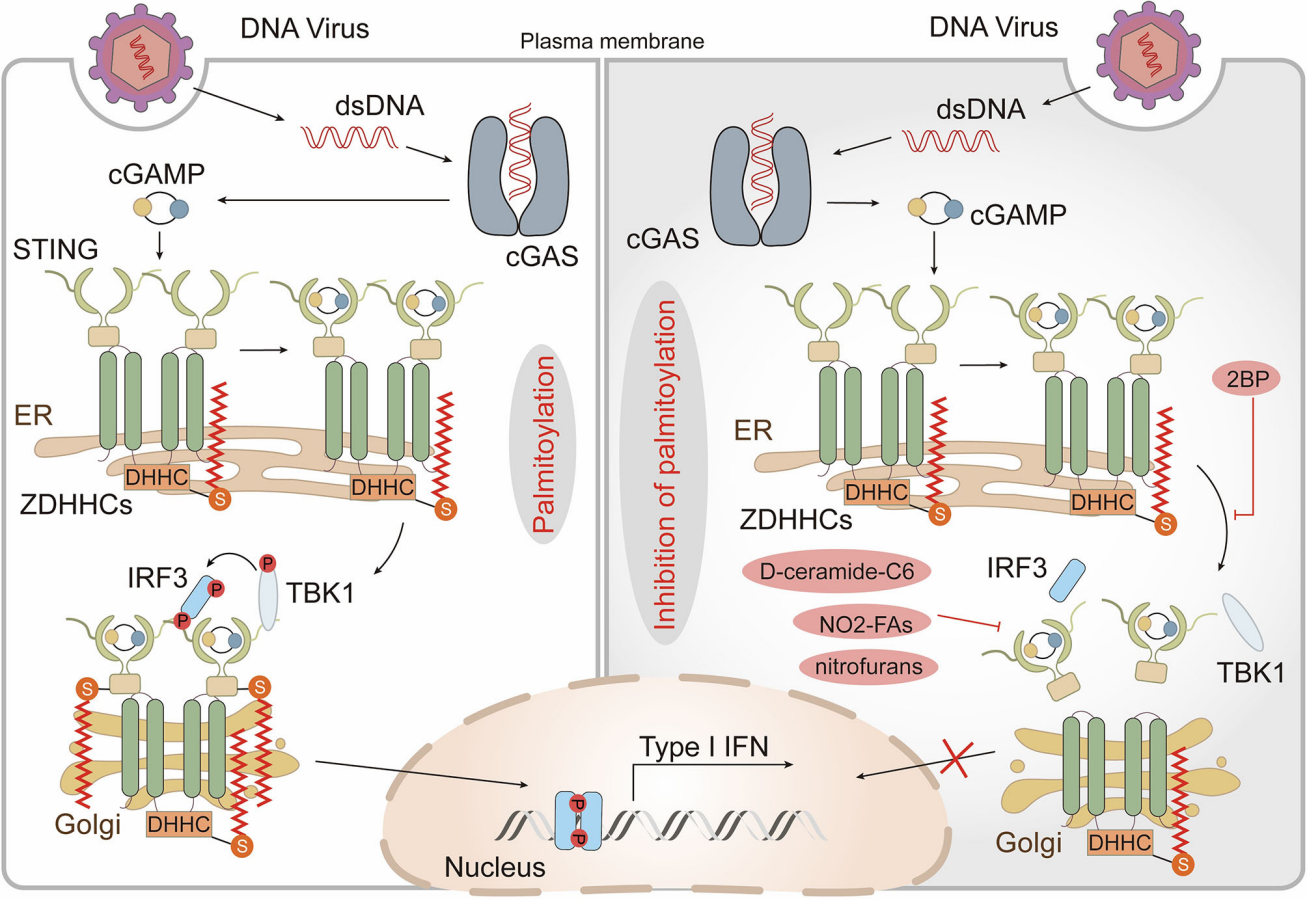
Palmitoylation in the cGAS-STING pathway. Cytoplasmic DNA recognized by cyclic GMP-AMP synthase (cGAS) triggers the production of the second messenger cyclic GMP–AMP (cGAMP), which binds and activates the cyclic-dinucleotide sensor, stimulator of interferon genes (STING). cGAMP promotes the translocation of STING from the endoplasmic reticulum (ER) to the Golgi where palmitoylation of the STING cytoplasmic proximal cysteine residues occurs. This facilitates the recruitment of TANK-binding kinase 1 (TBK1) and transcription factor interferon regulatory factor 3 (IRF3), triggers IRF3 nuclear entry and induces type I interferon (IFN) transcription. The non-specific Zinc finger aspartic acid-histidine-histidine-cysteine domain-containing protein (zDHHC)- palmitoyl acyltransferases (PATs) covalent inhibitor 2-bromopalmitate (2-BP) and the STING-selective palmitoylation inhibitors (nitro-fatty acids (NO2-FAs), nitrofurans) all block STING palmitoylation and inhibit production of type I IFN. D-ceramide-C6, which disrupts lipid rafts, inhibits the activation of TBK1 and IRF3 also.

Since all viruses possess genomes that differ from host nucleic acids and generate additional nucleic acid intermediates during replication, innate immune detection of viruses relies on the nucleic acid sensing system (93). In addition to several endosomal members of the TLR family, several cytoplasmic sensors [such as cyclic GMP-AMP synthase (cGAS) and retinoic acid-inducible protein I (RIG-I)] that detect DNA or RNA are also potent PRRs (93). In most cases, cytoplasmic DNA is recognized by cGAS which indirectly activates the intracellular stimulator of interferon genes (STING, also known as MPYS, ERIS, MITA, and TMEM173), triggering the production of anti-viral type I interferons (IFN-Is) (94–96). After binding to DNA, STING translocates from the ER to perinuclear compartments that include the Golgi, endosomes, and autophagy-related compartments (96, 97). Interestingly, STING-dependent signaling events, including phosphorylation of TANK-binding kinase 1 (TBK1) and transcription factor interferon regulatory factor 3 (IRF3) as well as induction of IFN-Is, can be blocked through disrupting ER to Golgi STING transport (98, 99). Thus, translocation of STING appears to be critical for its activation but the underlying mechanism is not yet known. Recently, it has been shown that activation of STING, in which palmitoylation plays a crucial role, occurs in the Golgi and is restricted to the trans-Golgi network (TGN). Treatment of emsCOS-1 cells, primary mouse embryonic fibroblasts (MEFs), and bone marrow-derived macrophages (BMDMs) with 2-BP effectively prevented agonist-induced STING palmitoylation. More importantly, 2-BP selectively inhibited STING-mediated cytosolic DNA sensing and its downstream interferon response. Furthermore, a Cys88/91Ser STING double mutant which had significantly reduced palmitoylation was unable to induce STING-dependent host defense gene expression and IFN-I responses upon infection with a DNA virus (99). Given that palmitoylation often localizes target proteins to lipid rafts (10), the authors speculated that STING functions in a similar manner. They treated cells with D-ceramide-C6 to disrupt lipid rafts on the Golgi apparatus and found that D-ceramide-C6 inhibited STING-dependent phosphorylation of TBK1 and IRF3. Based on these results, it was concluded that STING palmitoylation drives its aggregation in lipid rafts located in the TGN, which brings TBK1 and IRF3 into close proximity, promoting the IFN-I response (99) (**Figure 3**). zDHHC1 and 11 were identified as positive regulators of STING signaling but their palmitoyl transferase activity was not required for STING activation (100,) (101). By overexpressing individual zDHHC proteins in emsCOS-1 cells, investigators found that zDHHC3, 7 and 15 contribute to the palmitoylation of STING Cys88/91 (99). These findings suggested that palmitoylation is of great importance in the innate immune system response against DNA viruses.

### Palmitoylation and Interferon-Stimulated Genes

Activation of anti-virus PRRs triggers signaling cascades that stimulate the production of IFN-Is which induces the expression of a myriad of interferon-stimulated genes which leads to activation of the adaptive immune system and ultimately clearance of the infection (102). The IFNα/β receptor (IFNAR) is a heterodimer of IFNAR1 and IFNAR2, and both subunits can be palmitoylated. Palmitoylation of IFNAR1 occurs near the cytoplasmic end of the transmembrane domain, and is thought to determine the binding affinity of IFNAR1 for its downstream effector proteins. Absence of palmitoylation on IFNAR1 Cys463 selectively affects STAT2 activation, leading to incompetent STAT1 activation and nuclear translocation with a concurrent reduction in IFN-α-activated gene transcription. However, there is a paradox here in that although 2-BP treatment severely inhibits IFNAR1 endocytosis, the palmitoylation-deficient IFNAR1 mutant does not show any obvious endocytosis or intracellular distribution abnormalities (103). This might be due to the potential participation of other palmitoylated proteins such as IFNAR2. In addition, 2-BP is known to block the activities of a wide-range of cellular proteins including zDHHC-PATs which complicates the interpretation of data based solely on 2-BP (104).

Interferon-induced transmembrane proteins (IFITMs) are interferon inducible proteins that restrict viral infection and modulate membrane fluidity (105, 106). IFITM3 is located on the endosome that fuses with virus particles and enhances the trafficking of this pathogenic cargo to lysosomes. This property of IFITM3 is specific to certain viruses and requires its palmitoylation (107–109). The membrane proximal cysteine of IFITM3 can be palmitoylated and the palmitoylation site is highly conserved in vertebrates (110). It was initially thought that IFITM3 lysosomal localization and its antiviral activity were differentially regulated by palmitoylation and lysine ubiquitination. Specifically, palmitoylation was thought to enhance the membrane affinity and antiviral activity of IFITM3, while IFITM3 with ubiquitination causing the opposite effects (111). However, it is now known that IFITM3 activity is negatively regulated by at least three PTMs, including lysine ubiquitination, lysine methylation, and tyrosine phosphorylation. Up to now, only palmitoylation has been shown to have a positive effect on the antiviral activity of IFITM3 (112). IFITM3 is palmitoylated on three cysteine residues Cys71, 72 and 105 (113). To identify the zDHHC-PATs responsible for IFITM3 palmitoylation a library of human cell lines lacking one of zDHHCs1-24 was screened but it was found in the absence of any single zDHHC-PAT IFITM3 palmitoylation and its inhibitory effect on influenza virus infection remained strong. In contrast, in an overexpression screen more than half of all zDHHC-PATs were found to increase IFITM3 palmitoylation with zDHHC3, 7, 15 and 20 having the greatest effects. This suggests that multiple zDHHC-PATs can palmitoylate IFITM3 ensuring a strong antiviral response (114). IFITM proteins effectively limit infections by a range of viral pathogens including influenza A virus (IAV), Ebola virus, and severe acute respiratory syndrome coronavirus (SARS-COV) (108, 115). As other IFITM family members are associated with a diverse range of cellular activities including germ cell specification (IFITM1-IFITM3) (116, 117), osteoblast function, bone mineralization (IFITM5) (118), and adaptive immunity (IFITM1-3) (119), it is conceivable that palmitoylation may also be important for regulating these processes.

### Palmitoylation Regulates Tumor Necrosis Factor α-TNF Receptor 1 Signaling

**Figure 4:**
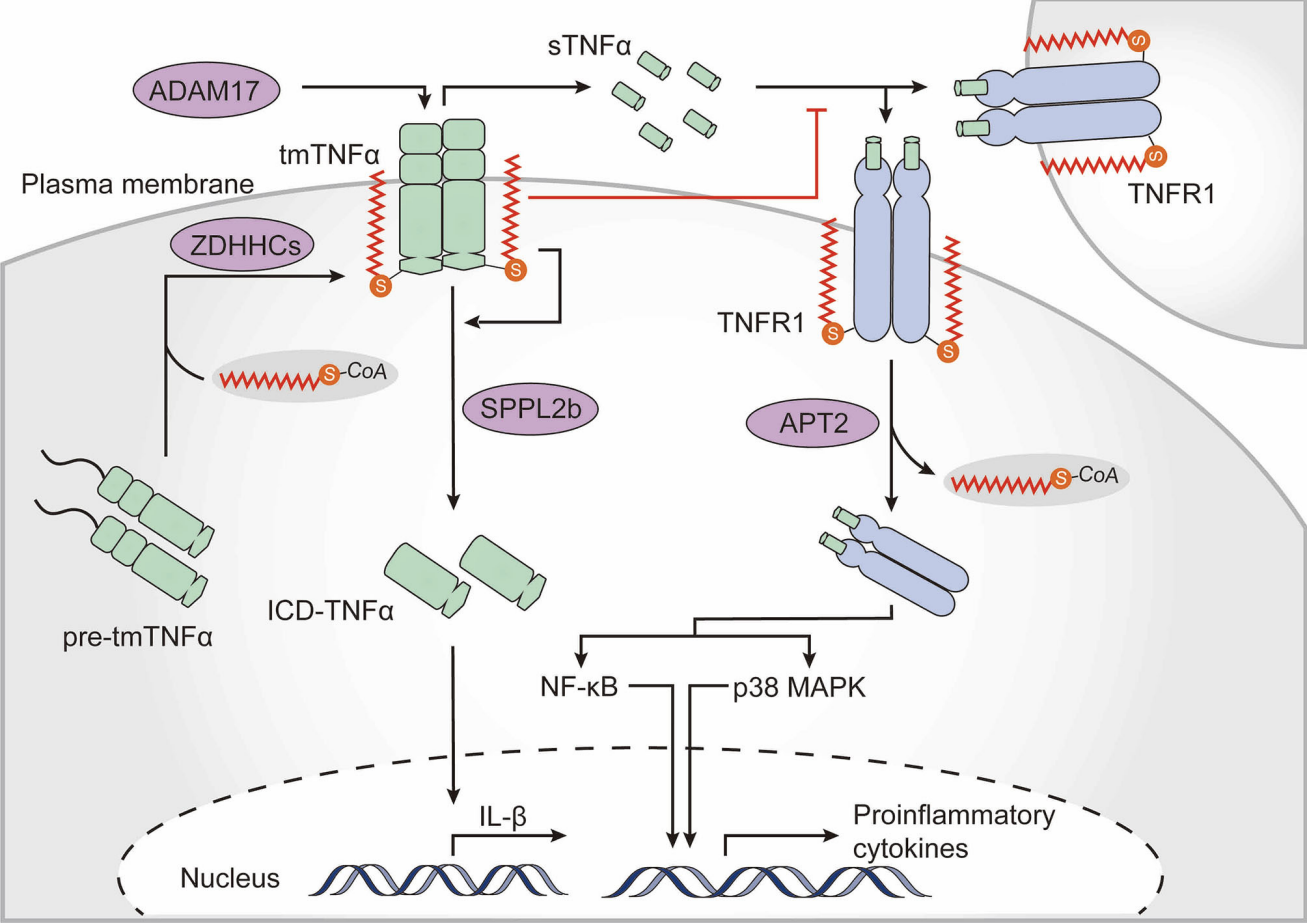
The role of palmitoylation in the TNFα-TNFR1 signaling pathway. Tumor necrosis factor α (TNFα) is palmitoylated at the boundary between its transmembrane and cytoplasmic domains, which is required for transmembrane TNFα (tmTNFα) localization to lipid rafts. After the extracellular domain of tmTNFα has been cleaved by the TNFα-converting enzyme disintegrin and metalloproteinase domain-containing protein 17 (ADAM17) and soluble TNFα (sTNFα) has been released, the palmitoylated raft-residing tmTNFα is further cleaved by signal peptide peptidase-like 2b (SPPL2b) to generate the intracellular domain of TNFα (ICD-TNFα), which activates the promoter of interleukin (IL)-1β. Binding of sTNFα to TNF receptor (TNFR) 1, however, is blocked by TNFα palmitoylation. TNFR1 also undergoes palmitoylation, which is required for localization within the plasma membrane. Upon sTNFα binding, TNFR1 is de-palmitoylated by acyl protein thioesterase 2 (APT2), which ultimately results in activation of nuclear factor kappa B (NF-κB) and p38-mitogen-activated protein kinase (MAPK).

Cytokines and their signaling networks are a multifunctional and tremendously important part of the innate immune system. TNFα is one of the most intensively studied pleiotropic pro-inflammatory cytokines and is primarily secreted by macrophages (120). Notably, TNFα is itself palmitoylated (121). Newly synthesized TNFα is a type II transmembrane 27-kDa precursor. Upon stimulation, the extracellular domain of transmembrane TNFα (tmTNFα) is cleaved by the TNFα-converting enzyme disintegrin and metalloproteinase domain-containing protein 17 (ADAM17), whereupon the biologically active soluble TNFα (sTNFα) is released to the extracellular milieu and activates multiple signaling pathways by interacting with TNF receptor (TNFR) 1 and TNFR2. In addition, the remaining membrane-bound TNFα fragment is further cleaved by the intramembrane protease signal peptide peptidase-like 2b (SPPL2b), whereupon the intracellular domain of TNFα (ICD-TNFα) is released, leading to the activation of the interleukin-1β (IL-1β) promoter (122, 123). Human tmTNFα is palmitoylated at Cys47 which is located at the boundary between the transmembrane and cytoplasmic domains (121). Palmitoylation does not substantially affect ADAM17-dependent cleavage of the ectodomain of tmTNFα but is required for localization of tmTNFα in lipid rafts and hinders binding of sTNFα to TNFR1. Moreover, palmitoylation of tmTNFα is also required for correct processing of SPPL2b-dependent cleavage. Palmitoylation-defective TNFα shows a rapid degradation of intracellular fragments, thus resulting in reduced ICD-TNFα production and IL-1β promoter activation (124) (**Figure 4**).

Upon binding to sTNFα, membrane-bound TNFR1 translocates to lipid rafts and activates NF-κB signaling (120). Palmitoylation of TNFR1 is required for its plasma membrane localization but the exact palmitoylation sites and the zDHHC-PATs are not yet known. Activated TNFR1 undergoes APT2-mediated depalmitoylation at Cys248 driving its translocation to intracellular signaling platforms, resulting in NF-κB activation (125) (**Figure 4**). These studies strongly suggest that palmitoylation of TNFα and TNFR is strongly related to regulation of the TNFα-induced signaling cascade. Of note, Sirtuin-6 (SIRT6) has been shown to regulate lysine fatty acylation of TNFα and promote TNFα secretion by defatty-acylation, which may reveal interesting connections between lysine fatty acylation and S-palmitoylation (126).

## Palmitoylation and Adaptive Immunity

The innate immune system provides rapid sensing and critical elimination of pathogens and leads to activation of the adaptive immune system, which has evolved to provide a broader and more finely tuned repertoire for recognizing and clearing infections. The adaptive immune system involves tightly regulated interplay between T and B lymphocytes and multiple adaptive immune effectors (127). Below, we discuss the current evidence for the involvement of palmitoylation in adaptive immunity.

### Palmitoylation Contributes to the Function of T Cell Co-Receptors

**Figure 5:**
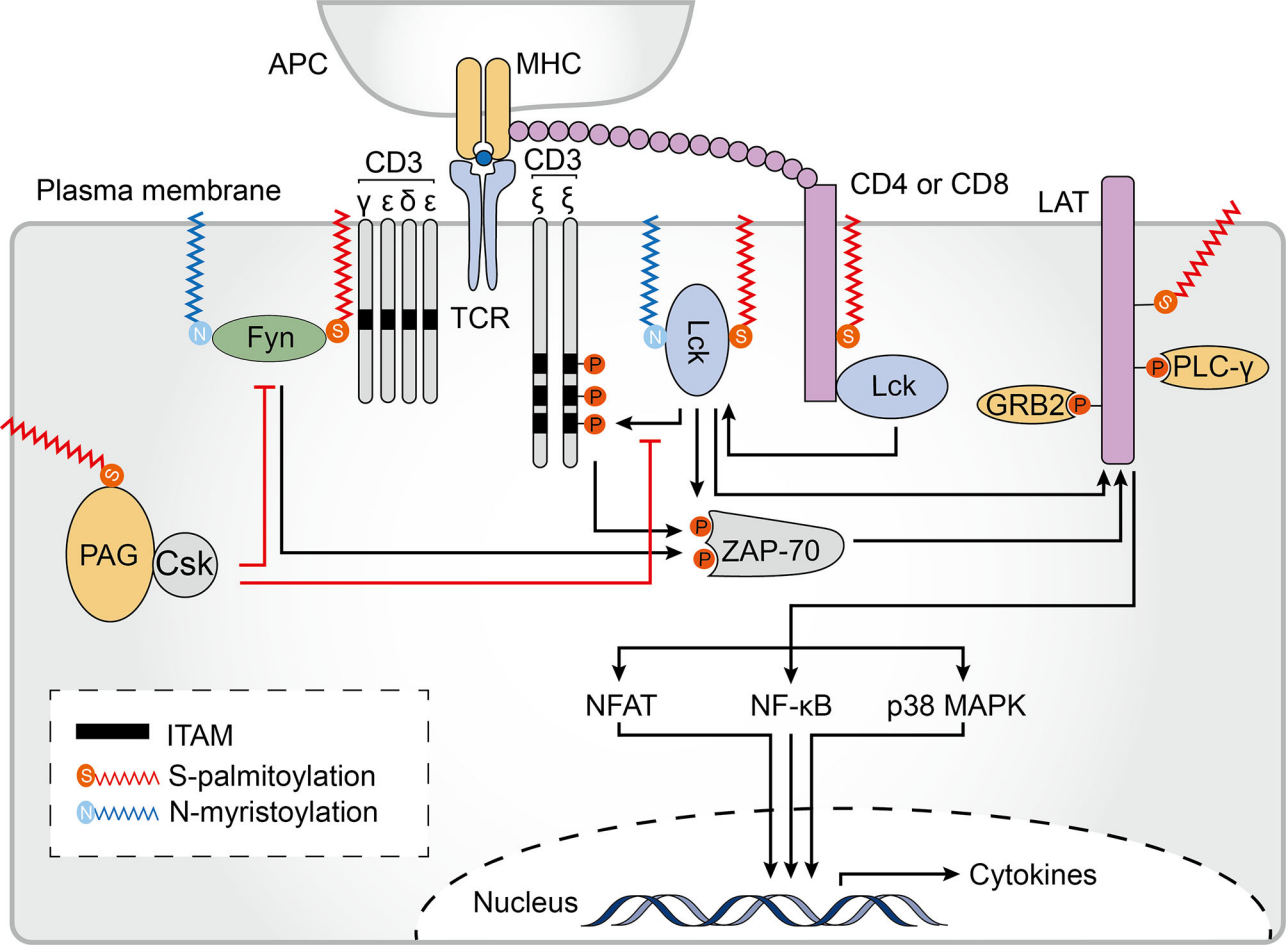
Palmitoylation in T cell signaling. Palmitoylation of CD4/CD8 is required for effective T cell receptor (TCR) signaling. Palmitoylation of CD4 contributes to its partitioning in lipid rafts. The tyrosine kinases Lck and Fyn both undergo N-myristoylation and palmitoylation, which is essential for their localization to the plasma membrane, and to lipid rafts in particular, and enhances the docking and activation of the zeta chain of T cell receptor associated protein kinase 70 (ZAP70). Palmitoylated and membrane localized linker for activation of T cells (LAT) is activated by ZAP-70 phosphorylation. Palmitoylation and phosphorylation of LAT generates binding sites for its downstream adaptors, including growth factor receptor bound protein 2 (GRB2) and phospholipase C gamma 1 (PLCγ1). These events are important for activating downstream TCR signaling pathways, including the nuclear factor of activated T-cells (NFAT), nuclear factor kappa B (NF-κB) and p38-mitogen-activated protein kinase (MAPK) pathways. Palmitoylation of phosphoprotein associated with glycosphingolipid-enriched microdomains (PAG) causes the tyrosine kinase Csk to be recruited to the plasma membrane where it acts as a negative regulator of T-cell signaling. ITAM, immune-receptor tyrosine-based activation motif; APC, antigen-presenting cells; MHC, major histocompatibility complex.

Pathogen-derived peptides associated with major histocompatibility complex (MHC) proteins are recognized by T cell receptors (TCRs), which perform essential functions in the initiation of the intracellular signals required for T cell activation and development. TCR engagement triggers recruitment of the co-stimulatory signaling receptors CD8 or CD4, which are transmembrane glycoproteins that increase T cell sensitivity to antigens and bind to the tyrosine kinase Lck as well as conserved regions in the MHC class I or II complexes (128). Despite the structures of CD4 and CD8 being quite distinct, they both contain palmitoylation sites (129, 130). Two cysteine residues, Cys394 and Cys397, at the juxtamembrane regions of CD4 can be palmitoylated (131). Palmitoylation of CD4 and binding to Lck contributes to its localization to lipid rafts, which is required for CD4-induced lipid raft aggregation, and contributes to the ability of CD4 to enhance receptor tyrosine phosphorylation and CD3 signaling (132) (**Figure 5**). Similarly, the localization of CD8 to lipid rafts is essential for its co-receptor function. Unlike CD4, which is a monomer, CD8 is a disulfide-linked heterodimer of CD8α and CD8β chains (129). Palmitoylation of the CD8β cytoplasmic tail in mice is required for efficient CD8 coreceptor function and is thought to endow CD8 with effective co-receptor function by coupling the TCR/CD3 with raft-associated CD8/Lck complexes (133, 134). Subsequent studies have shown that, in addition to human CD8β having two palmitoylation sites, human CD8α is also palmitoylated. Moreover, unlike what was described for mouse CD8, palmitoylation of human CD8 is not required for targeting to lipid rafts (130). Thus, there is only scarce knowledge about the significance and impact of CD4 and CD8 palmitoylation on T cell signaling. Additionally, the costimulatory molecules CD80 and CD86, which provide a second signal for full T-cell activation, are also palmitoylated in DCs (81). CD86 is palmitoylated primarily at the membrane-proximal Cys264 by DHHCs 2, 3, 6, and 7. However, whether palmitoylation of these molecules is involved in their trafficking to the DC surface, their subsequent clustering at the immunological synapse and impact on immune function remains to be elucidated. Obtaining a clearer picture of the possible engagement of these proteins in acquired immune responses remains a challenge for the future.

### Localization of Src Proteins Is Associated With Palmitoylation

Following TCR engagement by agonist peptides bound to MHC (pMHC) complexes, TCR-CD3 complexes cluster and recruit Lck, a Src family protein (135). A significant proportion of active Lck proteins reside outside of lipid rafts in resting T cells. On T cell activation, however, Lck translocates to lipid rafts, suggesting that Lck lipid raft localization may be important for TCR signaling (136, 137). To be precise, localization of Lck within lipid rafts first requires a N-myristoylation modification (after methionine removal, 14 carbon myristic acid is covalently attached to the N-terminus of the protein) which targets Lck to the plasma membrane. Subsequently, palmitoylation of Lck at Cys 3 and 5 by multiple zDHHC-PATs is required for a stable membrane-insertion of the protein (138–142) (**Figure 5**). A C3,5A double mutant (where Cys3 and Cys5 were both substituted with alanine) failed to localize to the plasma membrane and was unable promote downstream signaling in T lymphocytes (143). Of note, in most studies, Cys5 mutants have been found to have only a minimal reduction in membrane binding whereas the Cys3 mutants have a discernible reduction in the half-life of membrane binding, suggesting that single and dually palmitoylated Lcks may co-exist at the membrane (137, 138, 143, 144). Furthermore, a Lck C3,5 chimera which was anchored at the plasma membrane using a transmembrane domain instead of site-specific palmitoylation was unable to efficiently reconstitute TCR signaling, indicating that palmitoylation might play an important role in promoting TCR signaling beyond Lck membrane-binding (143).

Fyn is another highly expressed Src family tyrosine kinase that plays a role in T cell activation, and binds to a signaling motif contained in subunits of the TCR known as the immune-receptor tyrosine-based activation motif (ITAM) (145, 146). The Src homology (SH)-4 domain of Fyn contains 4 key amino acid residues, glycine 2, Cys3, lysine 7, and lysine 9, which are required for binding to ITAMs (145). Although these residues do not mediate direct contact with ITAMs, they are required for efficient self myristoylation and palmitoylation (147). Therefore, similarly to Lck, correct localization and function of Fyn is mediated by the combined effect of two lipid modifications, N-myristoylation and S-palmitoylation. However, in contrast to Lck, Fyn is mainly found within lipid rafts in resting T cells, and non-acylated Fyn can associate with membranes to some extent (60, 61). zDHHC2, 3, 7, 10, 15, 20 and 21 have been shown to palmitoylate Fyn *in vitro (* 48). Furthermore, although Cys3 and Cys6 can both be palmitoylated, only a minor fraction of Fyn is doubly palmitoylated with the majority of Fyn only singly palmitoylated at Cys3 (148), which is consistent with the importance of this palmitoylation site which has been shown to be associated with detergent insolubility and interaction with ITAMs (145, 147, 149).

### The Involvement of Palmitoylation in the Function of Transmembrane Adaptor Proteins

Transmembrane adaptor proteins (TRAPs) are structurally related proteins that enable the recruitment of intracellular effector molecules to the TCR complex (150). Lck activation and downstream phosphorylation of ITAMS leads to interaction between the zeta chain of the T cell receptor associated protein kinase 70 (ZAP70) and CD3ζ. Activated ZAP70 then phosphorylates TRAPs, which function as scaffold allowing for the subsequent recruitment of other adaptor molecules and initiation of a complex TCR-elicited signal transduction cascade (151). Among all TRAPs, the scaffolding transmembrane adaptor linker for activation of T cells (LAT) is a major target of activated ZAP70. Phosphorylated LAT recruits and activates growth factor receptor bound protein 2 (GRB2), phospholipase C gamma 1 (PLCγ1), phosphatidylinositol 3-kinase (PI3K), and other signaling molecules, leading to the signal propagation for T cell activation and function. At the junction of its transmembrane and cytoplasmic domains, LAT is palmitoylated at Cys26 and Cys29 with Cys26 being the most prominent site (152). The LAT transmembrane domain and palmitoylation at Cys26 are required for its stability and lipid raft localization (**Figure 5**). The absence of LAT palmitoylation results in remarkably reduced LAT expression levels and significantly suppresses its raft localization (153, 154). Moreover, LAT mutant displayed impaired recruitment and phosphorylation of both GRB2 and PLCγ1 as well as failed to propagate TCR-mediated signaling (155, 156). Based on these findings, lipid raft localization of LAT is thought to be required for effective TCR signaling. However, a chimeric LAT construct that was neither palmitoylated nor targeted to rafts could still function in TCR signaling, indicating that as long as LAT can recruit the appropriate downstream signaling molecules, it can function in or out of rafts during T cell activation (157). Interestingly, in antigen-specific anergic T cells, profoundly impaired LAT palmitoylation caused grossly reduced LAT recruitment to the immunological synapse, and prevented TCR/CD28-induced phosphorylation as well as downstream activation of PLCγ1 and PI3K, suggesting that dynamic palmitoylation of LAT also contributes to T cell anergy (158).

The phosphoprotein associated with glycosphingolipid-enriched microdomains (PAG) shares some degree of sequence homology with LAT and contains two palmitoylation sites at similar positions (159, 160). PAG acts as a negative regulator of T-cell signal propagation through the recruitment of C-terminal Src kinase (Csk) to the cell membrane which results in inactivation of Src kinases (161) (**Figure 5**). Although palmitoylation of PAG has not been studied extensively, it is already known that palmitoylation-deficient PAG has normal expression levels and is correctly targeted to the membrane but does not localize within lipid rafts nor block TCR-mediated proximal signaling events (162), suggesting that palmitoylated PAG may also be concentrated in lipid rafts. At present, there is still much to learn about the role of TRAP palmitoylation and raft localization.

### Palmitoylation in B Cell Signaling

Antibody production is a key feature of adaptive immunity and B cells are the only cell type that give rise to antibody-producing cells (163). B cell receptor (BCR) signaling is crucial for the initiation of B cell activation and fate decisions, as well as BCR-dependent antigen processing and presentation to T cells (164). The CD19/CD21/CD81 coreceptor complex can promote BCR signaling by prolonging the association of the BCR with lipid rafts (165, 166). Antigen ligation to the BCR-CD19/CD21/CD81 complex induces selective and rapid palmitoylation of CD81, which in turn prolongs the residency of BCR-CD19/CD21/CD81 in rafts, thereby amplifying raft clustering into larger domains with enhanced BCR-mediated signaling (167). Mutation of six CD81 in its juxtamembrane domain were required to completely eliminate detectable levels of palmitoylation, indicating that multiple sites in CD81 can be palmitoylated. In addition, palmitoylation-deficient CD81 mutant exhibited impaired association with CD81-interacting proteins CD9 and immunoglobulin superfamily member 8 (IGSF8), suggesting that the protein network surrounding CD81 would be affected when its palmitoylation ability is mutated (168). Moreover, palmitoylation of CD81 impaired recruitment of the epsilon isoform of 14-3-3, an intracellular signaling protein with a diverse array of binding partners regulating vital cellular processes, which may be dependent on cellular redox (169, 170).

A palmitoyl proteomic study in human B cells revealed over 100 palmitoylated proteins that are known to participate in a diverse range of cellular activities in B cells, including numerous MHC proteins, highlighting the important role that palmitoylation plays in regulating B cell behavior (12, 171). In particular, CD20 and CD23 display multiple interactions with immune effectors and are associated with cellular signaling cascades (171–174). CD20 which is only expressed in B cells and regulates B cell activation and proliferation (171, 175) is palmitoylated at Cys111 and Cys220 in its intracellular domain. CD23, as the low affinity receptor for immunoglobulin (Ig)E, is palmitoylated at Cys17 and Cys18 (174, 175). However, the functional importance and impact of CD20 and CD23 palmitoylation have not been explored. As palmitoylation has been investigated less in B compared to T cells, there is an urgent need to carry out further experiments to fully decipher the role of protein palmitoylation in B cell immune regulation.

### Palmitoylation in Fragment Crystallizable Receptor Signaling

Fc receptors (FcRs) play a crucial role recognizing antigens *via* Ig-mediated interactions and leading to diverse immune responses triggered by antigen recognition (176). Binding of the IgG complex to FcγR requires translocation of the receptor to lipid rafts which is dependent on its palmitoylation at Cys208 (177, 178). An FcγR Cys208Ala mutant had diminished translocation into lipid rafts and impaired FcγRII downstream signaling as measured by receptor phosphorylation and calcium mobilization (178).

In addition to FcRs themselves being palmitoylated themselves, their downstream effector proteins are also palmitoylated. ArfGAP with SH3 domain, ankyrin repeat and PH domain 2 (ASAP2), a scaffold protein involved in FcγR-mediated phagocytosis, is palmitoylated at Cys86 by the zDHHC6/Selenoprotein K (Selk) complex. Selk-dependent palmitoylation of ASAP2 is required for calpain-2 cleavage, which allows ASAP2 to leave the macrophage’s phagocytotic cups that are required for efficient uptake of immune complexes. Deletion of ASAP2 palmitoylation site impaired both maturation of the phagocytic cups and subsequent FcγR-mediated phagocytosis (179).

As with TCR and BCR, FcϵRI signal transduction requires Lyn activation and ITAM phosphorylation. Furthermore, myristoylation and palmitoylation of Lyn are essential for FcϵRI ITAM phosphorylation and FcϵRI-mediated signal transduction (180–182). Overall, palmitoylation plays a significant role in FcR signaling and related immune-events.

Although remarkable progress has been made, there is still much to be learned regarding the myriad of mechanisms by which protein palmitoylation regulates innate and adaptive immunity. Ongoing work in the field will continue to identify other proteins involved in immune signaling that get palmitoylated which will hopefully allow us to determine a complete picture of the palmitoylation regulome in immune cells.

## Palmitoylation and Immunologic Diseases

Recent advances in our understanding of the role that palmitoylation plays in regulating immune sensing and signaling have prompted deeper investigations in both preclinical and clinical contexts. Studies have already demonstrated that aberrant PAT activity and fluctuations in palmitoylation levels are associated with a large number of diverse pathological conditions (19). Recent findings related to the involvement of palmitoylation in human immunologic diseases are detailed in **Table 1**.

**Table 1:** Protein palmitoylation related immunologic diseases and therapies.

Target protein	Function of S-palmitoylation	Related diseases	Therapies	References
STING	membrane clustering	inflammatory and autoimmune diseases	pharmacological inhibitors (NO2-Fas, nitrofuran)	(183, 184)
NOD1/2	membrane association	immunologic and inflammatory diseases	DHHC covalent inhibitor 2-BP	(92)
MyD88	binding of IRAK4 to MyD88	sepsis	FASN inhibitor (C57)	(86)
PD-L1	ubiquitination blocking, lysosomal degradation suppressing	cancer	DHHC covalent inhibitor 2-BP, zDHHC3 knockdown, disruption of the zDHHC3/PD-L1 interaction with a peptide (S1-CPP)	(185)

### STING Palmitoylation as a Therapeutic Target for Inflammatory Diseases

Aberrant activation of the STING pathway has been implicated in the pathogenesis of a variety of inflammatory and autoimmune diseases including Aicardi–Goutières syndrome (ASG) and systemic lupus erythematosus (SLE) (186–188), which are both caused by loss of function mutations in the enzyme 3’ repair exonuclease I (TREX1) gene. Reduced TREX1 activity leads to increased levels of cytosolic dsDNA and ssDNA, which triggers hyperactivation of the cGAS-STING signaling pathway and results in a persistent release of pro-inflammatory cytokines (189–191). Gain-of-function mutations in the STING-encoding gene *TMEM173* maintains STING in an overactivated state which leads to a persistent “IFN signal”, and drives a systemic and debilitating inflammatory condition known as STING-associated vasculopathy with onset in infancy (SAVI) (192). The requirement of STING palmitoylation for its activation and signaling has prompted an intense search for potential inhibitors of STING palmitoylation. Pharmacological inhibitors of STING, which inhibit STING signaling by targeting its palmitoylation have recently been identified (183, 184). Endogenously formed nitro-fatty acids (NO2-FAs) inhibit STING signaling and prevent the release of IFN-Is in response to stimulation with STING agonists. Furthermore, NO2-FA treatment of immortalized fibroblasts from SAVI patients that had a gain-of-function mutation in STING dampened the STING-dependent IFN-I response. Mechanistically, NO2-FAs covalently modify STING through nitro-alkylation of Cys88 and Cys91, which abrogates STING palmitoylation inhibiting STING signaling (183). Furthermore, using a cell-based chemical screen, nitrofurans and their derivatives, reactive nitro-containing electrophiles, have been shown to block both STING palmitoylation and its signaling. Treatment of *Trex1^-/-^* mice with the nitrofuran compounds significantly reduced the serum levels of IFN-Is and ameliorated signs of systemic inflammation (184). Collectively, these two independent studies have shown that palmitoylation is a valid pharmacological target for the inhibition of STING signaling, which may represent a new and effective treatment strategy for STING-related autoinflammatory diseases.

### NOD Palmitoylation and NOD-Driven Inflammatory Diseases

Compelling evidence indicates that NOD1 and NOD2 polymorphisms are involved in a great number of immunologic and inflammatory diseases, including Crohn’s disease (CD), Blau syndrome, Behcet’s syndrome, ulcerative colitis (UC), atopic diseases, and early-onset sarcoidosis (EOS) (193, 194). As discussed previously, palmitoylation of NOD1 and NOD2 by zDHHC5 is required for proper membrane targeting and subsequent activation of NF-kB signaling upon detection of bacterial peptidoglycans (92). Notably, CD and EOS are associated with NOD2 palmitoylation. Several CD-associated NOD2 loss-of-function mutant proteins have significantly reduced palmitoylation levels which leads to more pronounced membrane dissociation and hence an inability to respond to muramyl dipeptide. Conversely, the EOS-associated NOD2 gain-of-function mutant has increased palmitoylation that drives NF-kB hyperactivity which can be prevented by 2-BP treatment (92). These findings suggest that there is a causal relationship between NOD2 (and potentially NOD1) palmitoylation and NOD-driven inflammatory diseases. The role that NOD palmitoylation plays in the pathogenesis of NOD-dependent autoimmune diseases needs to be further investigated and may eventually allow the development of new strategies to treat inflammatory and chronic bacterial infectious disorders.

### MyD88 Palmitoylation as a Therapeutic Niche for Controlling Sepsis

Sepsis, a common life-threatening systemic inflammation with a high mortality rate, is a serious threat to humans (195). Under septic conditions, the bactericidal activity of neutrophils upon bacterial infection is impaired due to the dysregulation of the host inflammatory signaling through TLR/MyD88, which involves p38MAPK activation and CXCR2 internalization (196). Thus, developing methods of suppressing the uncontrollable activation of TLR/MyD88 signaling may prevent host-mediated damage of patients with sepsis. As mentioned previously, MyD88 can be palmitoylated at Cys113 by zDHHC6 using endogenous fatty acids synthesized by FASN or exogenous fatty acids supplied by CD36-mediated uptake. C75, a FASN inhibitor, reduces internalization of surface CXCR2 and prevents desensitization of neutrophils which results in lower bacterial burden and better survival in septic mice. Moreover, *in vitro* treatment of neutrophils with C75 specifically blocked LPS-induced morphological changes and p38MAPK activation, which impaired neutrophil migration but did not affect IL-8- or N-formyl-methionyl-leucyl-phenylalanine (fMLP)-induced neutrophil activation (86). These studies suggest that FASN inhibitors may be useful therapeutic agents for the treatment of septic inflammation.

### Protein Palmitoylation in Anti-Tumor Immunity

Emerging evidence has linked specific palmitoylated proteins to carcinogenesis, cancer development, and therapy resistance. For example, programmed death ligand 1 (PD-L1), which is highly expressed in numerous human tumor cells, antagonizes T cell activation signals by binding to its receptor, programmed cell death 1(PD-1), on activated T cells (197). Checkpoint blockade therapy (which targets the interaction between PD-1 and PD-L1) promotes T cell-mediated tumor immune surveillance, and has achieved remarkable clinical efficacy in various cancers, including lung cancer, melanoma, bladder cancer, renal cell cancer, and colorectal cancer (198). PD-L1 is palmitoylated at Cys272 in its cytoplasmic domain. zDHHC3 palmitoylates and stabilizes PD-L1 in human colorectal cancer (CRC) cells by blocking PD-L1 ubiquitination thereby inhibiting endosomal sorting complexes required for transport (ESCRT)-mediated multivesicular body (MVB) sorting and lysosomal degradation, which results in elevated cell surface expression of PD-L1. Blockade of PD-L1 palmitoylation with 2BP, siRNA knockdown of zDHHC3 or a competitive, cell-penetrating disruptor peptide that prevented palmitoylation of PD-L1 by zDHHC3 promoted anti-tumor immunity *in vitro* and inhibited tumor growth *in vivo*, providing a new strategy for targeting PD-L1-dependent tumor immune escape (185). This study suggests that targeting PD-L1 palmitoylation may help to overcome PD-L1-mediated immune evasion in cancer. In the last few years, palmitoyl proteomic approaches have identified an array of mammalian palmitoylated proteins, many of which are cancer-related (199, 200). The growing list of palmitoylated cancer-related proteins includes those that function in anti-tumor immunity. Therefore, therapeutic strategies that modulate protein palmitoylation may prove to be effective for enhancing anti-tumor activity in people in the future.

## Conclusions and Perspectives

S-palmitoylation is a reversible PTM due to the labile nature of thioester bond and thus palmitoylation can dynamically and rapidly regulate protein conformation, protein stability, intracellular trafficking, membrane association of soluble proteins as well as protein-protein interactions (12). In this review, we have primarily focused on some classical and novel protein palmitoylation events in the immune system including pathogen sensing, immune molecule accumulation, anti-pathogen signal transduction, immune effector production and pro-inflammatory effects. In many cases, palmitoylation has been reported to target immune effectors to lipid rafts (124, 129, 132). However, the nature and function of lipid rafts is a controversial topic (201, 202). Recently, Lin has proposed that palmitoylation mediates targeting of both integral and peripheral membrane proteins to lipid rafts which promotes the formation of phase condensation in the membrane (26). The precise mechanism and function of palmitoylation in promoting immune protein membrane association and lipid raft localization is not yet fully understood. Targeting of palmitoylated immune effectors to the plasma membrane as well as to lipid rafts contributes to many signaling events, yet the functional impact of having such a large number of palmitoylated immune proteins is not yet fully appreciated.

The biomedical importance of PATs and APTs is underscored by their involvement in a variety of human diseases and this has aroused a growing interest in palmitoylation regulatory mechanisms (19, 203). Despite recent advances in understanding the mechanism and regulation of both PATs and APTs, many critical issues remain. One of the key issues is that the molecular basis of zDHHC-PAT substrate-specificity is currently poorly understood with no consensus palmitoylation motifs yet been identified. Moreover, some substrate proteins can be modified by multiple zDHHC-PATs, while others are mainly modified by one particular zDHHC-PAT (50–52, 204). Compared with the relatively large number of zDHHC-PATs that exist only a few APTs have been identified thus far. The mechanisms that govern the spatial distribution and organelle sorting of PATs and APTs also require deeper exploration. Of note, emerging evidence has identified interplay between palmitoylation and other PTMs such as ubiquitination and phosphorylation suggesting that immune regulation by palmitoylation may occur in conjunction with other PTMs (205–207). Deeper and wider knowledge concerning the mode of action of both PATs and APTs may help to develop therapeutic drugs that regulating the palmitoylation of specific target proteins.

Another important issue is how best to determine the sites of palmitoylation within a protein that is known to be acylated. Unlike myristoylation or isoprenylation, which have conserved lipid modification consensus sequence motifs, palmitoylation sites cannot yet be predicted on the amino acid sequence of the protein (208). Protein palmitoylation was originally studied using radioactive palmitate metabolic labeling methods. However, this approach is rarely used these days due to its limited sensitivity as well as safety concerns. The emergence of new technologies, such as acyl-biotinyl exchange and click-chemistry which now readily allow palmitoylation sites to be experimentally-determined has greatly promoted the study of lipid modifications.

When normal palmitoylation activity is dysregulated, serious consequences arise such as sensory incompetence or a hyper-response to pathogens, immune cell dysregulation, abnormally activated signaling pathways, inhibited protein degradation, and reduced apoptosis, which may lead to autoimmune diseases and tumor development (209). Thus, palmitoylation as a target for the treatment of these diseases has attracted increased attention. Looking ahead, one immediate goal is to develop selective zDHHC-PAT inhibitors as well as novel pharmacological APT protein inhibitors. Unfortunately, to date, no therapeutic drugs that regulate specific zDHHC-PATs have been developed. Unlike kinases where multiple inhibitors have been developed and tested in clinical trials the most commonly used palmitoylation inhibitor 2-BP cannot be used in the clinic due to its toxicity with off-target effects including interference with fatty acid metabolism (104). The depalmitoylation inhibitor Palm-B also blocks the activity of a series of serine hydrolases, limiting its clinical application (67). However, the selective manipulation of substrate recruitment to zDHHC-PATs may be therapeutically possible and would likely cause fewer side-effects (209). A feasible strategy for drug development is to screen chemical libraries consisting of 1000s of small molecules. This approach has been successfully used to identify nitrofuran and its derivatives as inhibitors of STING signaling and may be useful for treating autoimmune diseases. Furthermore, a high-throughput cell screen identified LGK974 as a competitive inhibitor of PORCN, an O-acyltransferase essential for palmitoylation and secretion of the Wnt ligand. LGK974 effectively inhibited Wnt signaling *in vitro* and *in vivo*, and played an active role in a variety of tumor models (210). In addition, inhibitors can be designed directly based on the latest crystal structure of palmitoyltransferases using a structure-oriented approach. Last, a competitive inhibitor, short peptide based on the sequence of PD-L1 has been shown to be effectively block palmitoylation of PD-L1 inducing its lysosomal clearance (185).

In summary, protein palmitoylation regulates numerous immune pathways and processes and may serve as a promising therapeutic target for aberrant immune diseases.

## Author Contributions

YZ, ZQ, and WS conceived and drafted the manuscript. YZ, ZQ, WS, and FC discussed the concepts of the manuscript. WS drew the figures. FZ approved the version to be submitted. All authors contributed to the article and approved the submitted version.

## Funding

This work was supported by the National Natural Science Foundation of China (31870902 to WS and 31871405 to FZ).

## Conflict of Interest

The authors declare that the research was conducted in the absence of any commercial or financial relationships that could be construed as a potential conflict of interest.

## Publisher’s Note

All claims expressed in this article are solely those of the authors and do not necessarily represent those of their affiliated organizations, or those of the publisher, the editors and the reviewers. Any product that may be evaluated in this article, or claim that may be made by its manufacturer, is not guaranteed or endorsed by the publisher.
